# Benzyl­tributyl­ammonium 4,6-dihydroxy­naphthalene-2-sulfonate

**DOI:** 10.1107/S1600536809000415

**Published:** 2009-01-17

**Authors:** Kazuya Uta, Jin Mizuguchi

**Affiliations:** a Department of Applied Physics, Graduate School of Engineering, Yokohama National University, 79-5 Tokiwadai, Hodogaya-ku, 240-8501 Yokohama, Japan

## Abstract

The title mol­ecular salt, C_19_H_34_N^+^·C_10_H_7_O_5_S^−^, is a charge-control agent used for toners in electrophotography with a high melting point of 508 K. In the crystal structure, the anions form inversion dimers, linked by pairs of O—H⋯O hydrogen bonds. Further O—H⋯O links between dimers generate anionic sheets propagating in (010). One of the *n*-butyl chains of the cation is disordered over two sets of sites in a 0.53:0.47 ratio.

## Related literature

For background on charge-control agents, see: Nash *et al.* (2001 ▶) and Uta *et al.* (2009 ▶). For the structures of benzyl­tributyl­ammonium 4-hydroxy­naphthalene-1-sulfonate, benzyl­tributyl­ammonium 6-hydroxy­naphthalene-2-sulfonate, benzyl­tributyl­ammonium 4-hydroxy­naphthalene-2-sulfonate and benzyl­tributyl­ammonium 7-hydroxy­naphthalene-1-sulfonate, see: Mizuguchi *et al.* (2007 ▶), Uta *et al.* (2009 ▶), Uta & Mizuguchi (2009 ▶) and Sato *et al.* (2009 ▶), respectively.
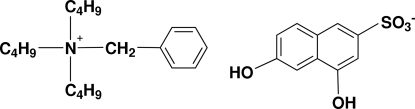


## Experimental

### 

#### Crystal data

C_19_H_34_N^+^·C_10_H_7_O_5_S^−^
*M* *_r_* = 515.70
Orthorhombic, 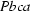

*a* = 18.6976 (3) Å
*b* = 15.3045 (2) Å
*c* = 19.7287 (3) Å
*V* = 5645.51 (14) Å^3^
*Z* = 8
Cu *K*α radiation
μ = 1.32 mm^−1^
*T* = 296.1 K
0.50 × 0.45 × 0.40 mm

#### Data collection

Rigaku R-AXIS RAPID diffractometer
Absorption correction: multi-scan (*ABSCOR*; Higashi, 1995 ▶) *T*_min_ = 0.425, *T*_max_ = 0.517 (expected range = 0.485–0.590)
49979 measured reflections
5149 independent reflections
2937 reflections with *F* ^2^ > 2σ(*F* ^2^)
*R*_int_ = 0.070

#### Refinement

*R*[*F* ^2^ > 2σ(*F* ^2^)] = 0.196
*wR*(*F* ^2^) = 0.547
*S* = 1.49
5149 reflections
332 parameters
H-atom parameters constrained
Δρ_max_ = 0.46 e Å^−3^
Δρ_min_ = −1.55 e Å^−3^

### 

Data collection: *PROCESS-AUTO* (Rigaku, 2006 ▶); cell refinement: *PROCESS-AUTO*; data reduction: *CrystalStructure* (Rigaku, 2006 ▶); program(s) used to solve structure: *SIR2004* (Burla *et al.*, 2003 ▶); program(s) used to refine structure: *SHELXL97* (Sheldrick, 2008 ▶); molecular graphics: *ORTEPIII* (Burnett & Johnson, 1996 ▶); software used to prepare material for publication: *CrystalStructure*.

## Supplementary Material

Crystal structure: contains datablocks global, I. DOI: 10.1107/S1600536809000415/hb2891sup1.cif

Structure factors: contains datablocks I. DOI: 10.1107/S1600536809000415/hb2891Isup2.hkl

Additional supplementary materials:  crystallographic information; 3D view; checkCIF report

## Figures and Tables

**Table 1:** Hydrogen-bond geometry (Å, °)

*D*—H⋯*A*	*D*—H	H⋯*A*	*D*⋯*A*	*D*—H⋯*A*
O4—H4*O*⋯O2^i^	0.82	1.94	2.758 (6)	172
O5—H5*O*⋯O3^ii^	0.82	1.87	2.623 (6)	153

Symmetry codes: (i) 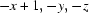; (ii) 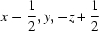.

**Fig. 1.**
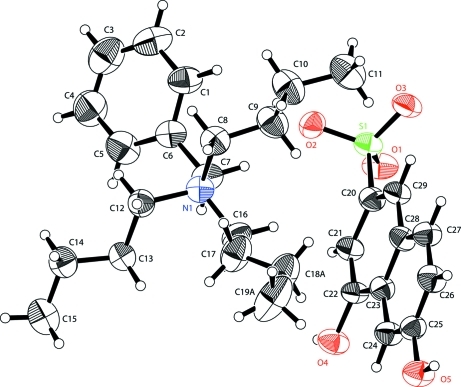
The asymmetric unit of (I), showing 30% probability displacement ellipsoids for the non-hydrogen atoms and only the major disorder component of the cation.

**Fig. 2.**
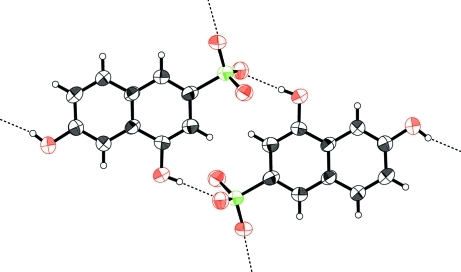
Inversion dimer unit connected by two O—H···O intermoleclar hydrogen bonds.

**Fig. 3.**
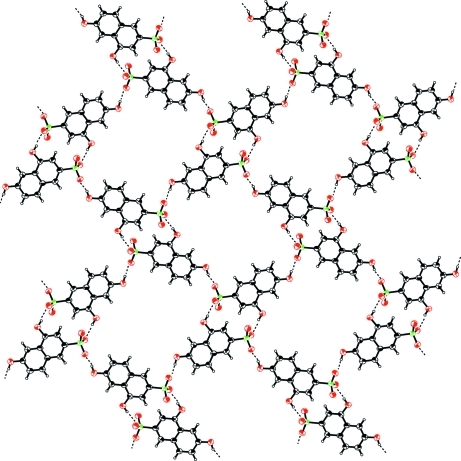
The two-dimensional hydrogen-bond network in the (010) plane.

## supplementary crystallographic information

### Comment

Compound (I) is a charge-control-agent used for toners in electrophotography. The background of the present study has been set out in our previous paper (Uta *et al.*, 2009). We have previously investigated the crystal structure of the following four isomers in connection with the mechanism of their high melting points: benzyltributylammonium 4-hydroxynaphthalene-1-sulfonate (Mizuguchi *et al.*, 2007), benzyltributylammonium 6-hydroxynaphthalene-2-sulfonate (Uta *et al.*, 2009, benzyltributylammonium 4-hydroxynaphthalene-2-sulfonate (Uta & Mizuguchi, 2009), and benzyltributylammonium 7-hydroxynaphthalene-1-sulfonate (Sato *et al.*, 2009). The melting points of these isomers are 462, 433, 451 and 439 K, respectively. Except for benzyltributylammonium 4-hydroxynaphthalene-2-sulfonate, the anions in the ammonium sulfates form chains of O—H···O intermolecular hydrogen bonds between the –OH group of one anion and the sulfonic O atom of the neighboring one. The present hydrogen-bond network is found to be responsible for the high thermal stability of these compounds. On the other hand, benzyltributylammonium 4-hydroxynaphthalene-2-sulfonate  which forms a two-dimensional hydrogen-bond network (Uta & Mizuguchi, 2009). The present paper deals with the structure of the title compound, (I), which includes two hydroxy groups in the naphthalene sulfonate (Fig. 1).

The ions in (I) have no crystallographically imposed symmetry. Fig. 2 shows a pseudo-dimer unit connected by O—H···O intermolecular hydrogen-bonded between the OH group of one anion and the sulfonic O atom of the neighboring one. Then, the dimer units consitute a two-dimensional hydrogen-bond network as shown in Fig. 3. There are four hydrogen bonds per molecule in the network, which contibutes to the high thermal stability of compound (I) as characterized by a melting point of 478 K.

### Experimental

The title compound was obtained from Orient Chemical Industries Ltd., and was recrystallized from an methanol solution. After 48 h, a number of colourless crystals were obtained in the form of blocks of (I).

### Refinement

C18 and C19 were found to be disordered over two sites each. The site occupancies for C18A/C18B and C19A/C19B are 0.53/0.47. These atoms were refined anisotropically. All H atoms were placed in geometrically idealized positions and constrained to ride on their parent atoms, with C—H = 0.93 Å (aromatic), 0.96 Å (methyl), or 0.97 Å (methylene), and O—H = 0.82 Å; *U*_iso_(H) = 1.2*U*_eq_(parent atom). The deepest hole is located 0.79Å from atom S1. The high *R* value of the present analysis can presumably be attributed to the poor crystallinity of the sample, although its size is sufficient.

### Crystal data

**Table:**

C_19_H_34_N^+^·C_10_H_7_O_5_S^−^	*F*(000) = 2224.00
*M**_r_* = 515.70	*D*_x_ = 1.214 Mg m^−^^3^
Orthorhombic, *P**b**c**a*	Cu *K*α radiation, λ = 1.54187 Å
Hall symbol: -P 2ac 2ab	Cell parameters from 29523 reflections
*a* = 18.6976 (3) Å	θ = 3.3–68.2°
*b* = 15.3045 (2) Å	µ = 1.32 mm^−^^1^
*c* = 19.7287 (3) Å	*T* = 296 K
*V* = 5645.51 (14) Å^3^	Block, colourless
*Z* = 8	0.50 × 0.45 × 0.40 mm

### Data collection

**Table:**

Rigaku R-AXIS RAPID diffractometer	2937 reflections with *F*^2^ > 2σ(*F*^2^)
Detector resolution: 10.00 pixels mm^-1^	*R*_int_ = 0.070
ω scans	θ_max_ = 68.2°
Absorption correction: multi-scan (*ABSCOR*; Higashi, 1995)	*h* = −22→22
*T*_min_ = 0.425, *T*_max_ = 0.517	*k* = −18→18
49979 measured reflections	*l* = −23→23
5149 independent reflections	

### Refinement

**Table:**

Refinement on *F*^2^	H-atom parameters constrained
*R*[*F*^2^ > 2σ(*F*^2^)] = 0.196	*w* = 1/[σ^2^(*F*_o_^2^) + (0.3*P*)^2^] where *P* = (*F*_o_^2^ + 2*F*_c_^2^)/3
*wR*(*F*^2^) = 0.547	(Δ/σ)_max_ < 0.001
*S* = 1.49	Δρ_max_ = 0.46 e Å^−^^3^
5149 reflections	Δρ_min_ = −1.55 e Å^−^^3^
332 parameters	

### Special details

**Table:**

Geometry. All e.s.d.'s (except the e.s.d. in the dihedral angle between two l.s. planes) are estimated using the full covariance matrix. The cell e.s.d.'s are taken into account individually in the estimation of e.s.d.'s in distances, angles and torsion angles; correlations between e.s.d.'s in cell parameters are only used when they are defined by crystal symmetry. An approximate (isotropic) treatment of cell e.s.d.'s is used for estimating e.s.d.'s involving l.s. planes.
Refinement. Refinement of *F*^2^ against ALL reflections. The weighted *R*-factor *wR* and goodness of fit *S* are based on *F*^2^, conventional *R*-factors *R* are based on *F*, with *F* set to zero for negative *F*^2^. The threshold expression of *F*^2^ > σ(*F*^2^) is used only for calculating *R*-factors(gt) *etc*. and is not relevant to the choice of reflections for refinement. *R*-factors based on *F*^2^ are statistically about twice as large as those based on *F*, and *R*- factors based on ALL data will be even larger.

### Fractional atomic coordinates and isotropic or equivalent isotropic displacement parameters (Å^2^)

**Table:**

	*x*	*y*	*z*	*U*_iso_*/*U*_eq_	Occ. (<1)
S1	0.57640 (9)	−0.08254 (12)	0.11259 (8)	0.0863 (7)	
O1	0.5860 (2)	−0.1387 (3)	0.0538 (2)	0.1069 (16)	
O2	0.6038 (2)	0.0070 (3)	0.1008 (2)	0.0958 (13)	
O3	0.6031 (2)	−0.1202 (3)	0.1753 (2)	0.0985 (14)	
O4	0.3225 (2)	−0.0388 (3)	0.0178 (2)	0.1020 (14)	
O5	0.1550 (2)	−0.0580 (3)	0.2110 (2)	0.1031 (14)	
N1	0.4640 (2)	0.2386 (3)	0.1198 (2)	0.0886 (15)	
C1	0.6461 (4)	0.2299 (6)	0.1023 (4)	0.120 (2)	
C2	0.7111 (5)	0.2710 (8)	0.0881 (6)	0.141 (3)	
C3	0.7168 (7)	0.3210 (8)	0.0321 (7)	0.161 (4)	
C4	0.6604 (6)	0.3332 (8)	−0.0113 (7)	0.157 (3)	
C5	0.5955 (5)	0.2916 (7)	0.0026 (5)	0.134 (3)	
C6	0.5872 (3)	0.2396 (5)	0.0600 (4)	0.098 (2)	
C7	0.5181 (3)	0.1910 (5)	0.0727 (3)	0.098 (2)	
C8	0.4935 (4)	0.2484 (5)	0.1924 (3)	0.100 (2)	
C9	0.5092 (5)	0.1646 (6)	0.2294 (3)	0.121 (2)	
C10	0.5168 (6)	0.1769 (6)	0.3031 (4)	0.134 (3)	
C11	0.5276 (6)	0.0902 (6)	0.3404 (5)	0.144 (3)	
C12	0.4475 (4)	0.3312 (4)	0.0937 (3)	0.0960 (19)	
C13	0.4049 (4)	0.3341 (4)	0.0280 (4)	0.105 (2)	
C14	0.4016 (5)	0.4253 (5)	0.0017 (4)	0.117 (2)	
C15	0.3534 (6)	0.4327 (7)	−0.0593 (5)	0.146 (3)	
C16	0.3975 (3)	0.1816 (5)	0.1193 (4)	0.102 (2)	
C17	0.3383 (4)	0.2173 (5)	0.1649 (5)	0.136 (3)	
C18A	0.2950 (11)	0.1572 (10)	0.1998 (15)	0.136 (3)	0.53
C18B	0.2741 (8)	0.1655 (15)	0.1599 (12)	0.123 (6)	0.47
C19A	0.2290 (14)	0.2034 (18)	0.230 (2)	0.190 (7)	0.53
C19B	0.2168 (17)	0.163 (2)	0.217 (2)	0.190 (7)	0.47
C20	0.4837 (3)	−0.0710 (4)	0.1227 (3)	0.0823 (17)	
C21	0.4407 (3)	−0.0583 (4)	0.0648 (3)	0.0859 (16)	
C22	0.3669 (3)	−0.0524 (4)	0.0723 (3)	0.0809 (15)	
C23	0.3341 (3)	−0.0600 (3)	0.1368 (2)	0.0770 (14)	
C24	0.2586 (3)	−0.0569 (4)	0.1444 (3)	0.0860 (17)	
C25	0.2293 (3)	−0.0607 (4)	0.2068 (3)	0.0840 (16)	
C26	0.2706 (4)	−0.0672 (4)	0.2650 (3)	0.0964 (19)	
C27	0.3450 (3)	−0.0732 (4)	0.2580 (3)	0.0898 (18)	
C28	0.3771 (3)	−0.0692 (4)	0.1947 (2)	0.0793 (15)	
C29	0.4536 (3)	−0.0750 (4)	0.1870 (3)	0.0824 (16)	
H1	0.6421	0.1954	0.1409	0.144*	
H2	0.7500	0.2639	0.1170	0.169*	
H3	0.7603	0.3480	0.0227	0.193*	
H4	0.6653	0.3686	−0.0494	0.188*	
H4O	0.3466	−0.0336	−0.0167	0.122*	
H5	0.5572	0.2987	−0.0270	0.161*	
H5O	0.1428	−0.0617	0.2508	0.124*	
H7A	0.5294	0.1347	0.0925	0.118*	
H7B	0.4952	0.1802	0.0294	0.118*	
H8A	0.5372	0.2824	0.1903	0.119*	
H8B	0.4592	0.2816	0.2188	0.119*	
H9A	0.4709	0.1234	0.2208	0.145*	
H9B	0.5531	0.1397	0.2116	0.145*	
H10A	0.4742	0.2054	0.3205	0.160*	
H10B	0.5573	0.2148	0.3119	0.160*	
H11A	0.4981	0.0461	0.3202	0.217*	
H11B	0.5147	0.0972	0.3872	0.217*	
H11C	0.5769	0.0731	0.3373	0.217*	
H12A	0.4210	0.3624	0.1284	0.115*	
H12B	0.4923	0.3619	0.0868	0.115*	
H13A	0.4274	0.2966	−0.0055	0.126*	
H13B	0.3569	0.3125	0.0360	0.126*	
H14A	0.4493	0.4444	−0.0105	0.140*	
H14B	0.3841	0.4636	0.0372	0.140*	
H15A	0.3689	0.3924	−0.0935	0.218*	
H15B	0.3554	0.4912	−0.0768	0.218*	
H15C	0.3051	0.4192	−0.0463	0.218*	
H16A	0.4101	0.1233	0.1343	0.123*	
H16B	0.3796	0.1773	0.0733	0.123*	
H17A	0.3603	0.2553	0.1983	0.163*	0.53
H17B	0.3073	0.2534	0.1372	0.163*	0.53
H17C	0.3547	0.2175	0.2116	0.163*	0.47
H17D	0.3277	0.2771	0.1521	0.163*	0.47
H18A	0.3224	0.1302	0.2359	0.163*	0.53
H18B	0.2796	0.1116	0.1690	0.163*	0.53
H18C	0.2891	0.1056	0.1525	0.148*	0.47
H18D	0.2497	0.1839	0.1189	0.148*	0.47
H19A	0.2411	0.2277	0.2733	0.284*	0.53
H19B	0.1909	0.1620	0.2352	0.284*	0.53
H19C	0.2140	0.2494	0.2000	0.284*	0.53
H19D	0.2180	0.2174	0.2419	0.284*	0.47
H19E	0.2269	0.1158	0.2476	0.284*	0.47
H19F	0.1702	0.1554	0.1977	0.284*	0.47
H21	0.4614	−0.0538	0.0221	0.103*	
H24	0.2294	−0.0523	0.1065	0.103*	
H26	0.2494	−0.0675	0.3076	0.116*	
H27	0.3732	−0.0800	0.2964	0.108*	
H29	0.4826	−0.0815	0.2249	0.099*	

### Atomic displacement parameters (Å^2^)

**Table:**

	*U*^11^	*U*^22^	*U*^33^	*U*^12^	*U*^13^	*U*^23^
S1	0.0805 (10)	0.1074 (13)	0.0711 (11)	0.0042 (7)	−0.0076 (6)	−0.0044 (7)
O1	0.091 (2)	0.143 (4)	0.087 (2)	0.019 (2)	−0.004 (2)	−0.046 (2)
O2	0.091 (2)	0.104 (3)	0.092 (2)	−0.019 (2)	0.003 (2)	0.011 (2)
O3	0.084 (2)	0.134 (3)	0.077 (2)	0.004 (2)	−0.015 (2)	0.015 (2)
O4	0.090 (2)	0.151 (4)	0.065 (2)	−0.001 (2)	−0.004 (2)	0.012 (2)
O5	0.076 (2)	0.145 (3)	0.088 (3)	0.001 (2)	0.008 (2)	0.002 (2)
N1	0.089 (3)	0.095 (3)	0.082 (3)	0.004 (2)	0.003 (2)	−0.010 (2)
C1	0.108 (5)	0.152 (7)	0.100 (5)	−0.010 (5)	−0.010 (4)	−0.016 (4)
C2	0.105 (5)	0.191 (10)	0.127 (7)	−0.024 (6)	0.005 (5)	−0.034 (7)
C3	0.130 (8)	0.159 (9)	0.195 (13)	−0.013 (7)	0.046 (9)	−0.017 (9)
C4	0.126 (7)	0.178 (9)	0.165 (9)	−0.004 (7)	0.033 (7)	0.033 (8)
C5	0.124 (6)	0.173 (8)	0.106 (6)	0.012 (6)	0.019 (5)	0.017 (6)
C6	0.090 (4)	0.110 (5)	0.094 (4)	0.007 (3)	0.004 (3)	−0.020 (3)
C7	0.084 (3)	0.117 (5)	0.093 (4)	0.005 (3)	0.010 (3)	−0.021 (3)
C8	0.114 (5)	0.107 (4)	0.077 (4)	0.004 (3)	0.004 (3)	−0.016 (3)
C9	0.150 (7)	0.128 (6)	0.085 (5)	0.017 (5)	−0.010 (4)	−0.004 (4)
C10	0.176 (8)	0.128 (6)	0.097 (6)	0.036 (6)	−0.006 (5)	−0.006 (4)
C11	0.155 (8)	0.176 (9)	0.102 (6)	0.032 (6)	0.013 (6)	0.008 (5)
C12	0.096 (4)	0.095 (4)	0.097 (4)	0.000 (3)	0.001 (3)	0.004 (3)
C13	0.110 (4)	0.102 (4)	0.104 (5)	0.000 (4)	−0.009 (4)	0.013 (3)
C14	0.125 (6)	0.117 (5)	0.108 (5)	0.002 (4)	0.004 (5)	0.013 (4)
C15	0.161 (9)	0.136 (7)	0.140 (8)	0.023 (6)	0.003 (7)	0.018 (6)
C16	0.081 (3)	0.108 (5)	0.119 (6)	−0.012 (3)	0.011 (3)	−0.012 (4)
C17	0.116 (5)	0.117 (5)	0.175 (8)	−0.009 (4)	0.052 (5)	−0.006 (5)
C18A	0.116 (5)	0.117 (5)	0.175 (8)	−0.009 (4)	0.052 (5)	−0.006 (5)
C18B	0.097 (10)	0.158 (15)	0.114 (13)	−0.013 (9)	−0.007 (8)	−0.011 (11)
C19A	0.150 (11)	0.131 (17)	0.288 (18)	−0.026 (10)	0.112 (12)	−0.067 (15)
C19B	0.150 (11)	0.131 (17)	0.288 (18)	−0.026 (10)	0.112 (12)	−0.067 (15)
C20	0.092 (4)	0.096 (3)	0.059 (3)	−0.003 (3)	−0.007 (2)	−0.001 (2)
C21	0.084 (3)	0.101 (3)	0.074 (3)	0.004 (3)	−0.008 (3)	−0.003 (3)
C22	0.069 (3)	0.104 (4)	0.070 (3)	−0.002 (3)	−0.012 (2)	−0.002 (2)
C23	0.085 (3)	0.092 (3)	0.054 (2)	−0.006 (2)	0.002 (2)	0.003 (2)
C24	0.071 (3)	0.113 (4)	0.074 (3)	0.002 (3)	0.000 (2)	0.004 (3)
C25	0.075 (3)	0.098 (4)	0.079 (3)	−0.010 (3)	0.001 (2)	0.002 (3)
C26	0.105 (4)	0.122 (5)	0.062 (3)	0.001 (3)	0.005 (3)	0.005 (3)
C27	0.082 (3)	0.122 (5)	0.065 (3)	−0.007 (3)	0.002 (2)	0.000 (3)
C28	0.086 (3)	0.096 (3)	0.055 (2)	0.009 (3)	−0.001 (2)	0.003 (2)
C29	0.075 (3)	0.103 (4)	0.069 (3)	0.003 (2)	−0.009 (2)	0.001 (2)

### Geometric parameters (Å, °)

**Table:**

S1—O1	1.455 (5)	C4—H4	0.930
S1—O2	1.481 (5)	C5—H5	0.930
S1—O3	1.453 (4)	C7—H7A	0.970
S1—C20	1.753 (7)	C7—H7B	0.970
O4—C22	1.375 (7)	C8—H8A	0.970
O5—C25	1.392 (7)	C8—H8B	0.970
N1—C7	1.555 (9)	C9—H9A	0.970
N1—C8	1.543 (8)	C9—H9B	0.970
N1—C12	1.540 (9)	C10—H10A	0.970
N1—C16	1.519 (9)	C10—H10B	0.970
C1—C2	1.398 (14)	C11—H11A	0.960
C1—C6	1.388 (11)	C11—H11B	0.960
C2—C3	1.348 (19)	C11—H11C	0.960
C3—C4	1.372 (19)	C12—H12A	0.970
C4—C5	1.399 (16)	C12—H12B	0.970
C5—C6	1.394 (13)	C13—H13A	0.970
C6—C7	1.511 (10)	C13—H13B	0.970
C8—C9	1.504 (11)	C14—H14A	0.970
C9—C10	1.473 (11)	C14—H14B	0.970
C10—C11	1.530 (13)	C15—H15A	0.960
C12—C13	1.522 (11)	C15—H15B	0.960
C13—C14	1.491 (10)	C15—H15C	0.960
C14—C15	1.507 (14)	C16—H16A	0.970
C16—C17	1.527 (12)	C16—H16B	0.970
C17—C18A	1.40 (2)	C17—H17A	0.970
C17—C18B	1.44 (2)	C17—H17B	0.970
C18A—C19A	1.54 (3)	C17—H17C	0.970
C18B—C19B	1.56 (4)	C17—H17D	0.970
C20—C21	1.411 (9)	C18A—H18A	0.970
C20—C29	1.389 (8)	C18A—H18B	0.970
C21—C22	1.389 (8)	C18B—H18C	0.970
C22—C23	1.416 (8)	C18B—H18D	0.970
C23—C24	1.421 (8)	C19A—H19A	0.960
C23—C28	1.405 (8)	C19A—H19B	0.960
C24—C25	1.347 (9)	C19A—H19C	0.960
C25—C26	1.387 (9)	C19B—H19D	0.960
C26—C27	1.401 (10)	C19B—H19E	0.960
C27—C28	1.387 (8)	C19B—H19F	0.960
C28—C29	1.440 (9)	C21—H21	0.930
O4—H4O	0.820	C24—H24	0.930
O5—H5O	0.820	C26—H26	0.930
C1—H1	0.930	C27—H27	0.930
C2—H2	0.930	C29—H29	0.930
C3—H3	0.930		
			
O2···O4^i^	2.758 (6)	O5···H12A^vi^	2.482
O3···O5^ii^	2.623 (6)	O5···H17A^vi^	2.883
O4···O2^i^	2.758 (6)	O5···H17D^vi^	2.797
O5···O3^iii^	2.623 (6)	C2···H19A^ii^	2.869
S1···H4O^i^	2.967	C4···H18D^vii^	2.713
S1···H5O^ii^	2.984	C7···H21^i^	2.837
O1···H3^iv^	2.946	C10···H19F^ii^	2.888
O1···H13A^i^	2.609	C11···H19F^ii^	2.944
O1···H7B^i^	2.323	C21···H7B^i^	2.895
O1···H16B^i^	2.655	C23···H18B	2.888
O2···H4O^i^	1.943	C23···H18C	2.688
O2···H21^i^	2.807	C24···H18B	2.653
O2···H7A	2.403	C24···H18C	2.556
O3···H5O^ii^	1.865	C25···H17D^vi^	2.909
O3···H8B^v^	2.825	C25···H18B	2.897
O3···H26^ii^	2.871	C25···H18C	2.979
O4···H15C^vi^	2.777	C25···H19E	2.820
O5···H11C^iii^	2.659	C26···H19E	2.937
			
O1—S1—O2	112.3 (2)	C9—C10—H10A	109.2
O1—S1—O3	113.7 (3)	C9—C10—H10B	109.2
O1—S1—C20	105.8 (2)	C11—C10—H10A	109.2
O2—S1—O3	112.4 (2)	C11—C10—H10B	109.2
O2—S1—C20	105.4 (2)	H10A—C10—H10B	107.9
O3—S1—C20	106.4 (2)	C10—C11—H11A	109.5
C7—N1—C8	111.6 (5)	C10—C11—H11B	109.5
C7—N1—C12	111.2 (5)	C10—C11—H11C	109.5
C7—N1—C16	105.2 (5)	H11A—C11—H11B	109.5
C8—N1—C12	107.0 (5)	H11A—C11—H11C	109.5
C8—N1—C16	110.8 (5)	H11B—C11—H11C	109.5
C12—N1—C16	111.3 (5)	N1—C12—H12A	108.6
C2—C1—C6	121.4 (8)	N1—C12—H12B	108.6
C1—C2—C3	119.3 (10)	C13—C12—H12A	108.6
C2—C3—C4	121.9 (11)	C13—C12—H12B	108.6
C3—C4—C5	118.9 (11)	H12A—C12—H12B	107.6
C4—C5—C6	121.0 (9)	C12—C13—H13A	109.6
C1—C6—C5	117.5 (7)	C12—C13—H13B	109.6
C1—C6—C7	121.7 (7)	C14—C13—H13A	109.6
C5—C6—C7	120.6 (7)	C14—C13—H13B	109.6
N1—C7—C6	115.1 (6)	H13A—C13—H13B	108.1
N1—C8—C9	115.9 (6)	C13—C14—H14A	109.2
C8—C9—C10	112.9 (7)	C13—C14—H14B	109.2
C9—C10—C11	112.2 (7)	C15—C14—H14A	109.2
N1—C12—C13	114.5 (5)	C15—C14—H14B	109.2
C12—C13—C14	110.2 (6)	H14A—C14—H14B	107.9
C13—C14—C15	111.9 (7)	C14—C15—H15A	109.5
N1—C16—C17	112.7 (6)	C14—C15—H15B	109.5
C16—C17—C18A	118.2 (10)	C14—C15—H15C	109.5
C16—C17—C18B	111.5 (11)	H15A—C15—H15B	109.5
C17—C18A—C19A	110.4 (15)	H15A—C15—H15C	109.5
C17—C18B—C19B	122 (2)	H15B—C15—H15C	109.5
S1—C20—C21	119.1 (4)	N1—C16—H16A	109.1
S1—C20—C29	120.1 (4)	N1—C16—H16B	109.1
C21—C20—C29	120.8 (6)	C17—C16—H16A	109.1
C20—C21—C22	119.3 (5)	C17—C16—H16B	109.1
O4—C22—C21	121.7 (5)	H16A—C16—H16B	107.8
O4—C22—C23	117.0 (5)	C16—C17—H17A	107.8
C21—C22—C23	121.3 (5)	C16—C17—H17B	107.8
C22—C23—C24	121.5 (5)	C16—C17—H17C	109.3
C22—C23—C28	119.4 (5)	C16—C17—H17D	109.3
C24—C23—C28	119.0 (5)	C18A—C17—H17A	107.8
C23—C24—C25	120.0 (5)	C18A—C17—H17B	107.8
O5—C25—C24	117.3 (5)	C18B—C17—H17C	109.3
O5—C25—C26	120.5 (5)	C18B—C17—H17D	109.3
C24—C25—C26	122.2 (6)	H17A—C17—H17B	107.1
C25—C26—C27	118.4 (6)	H17C—C17—H17D	108.0
C26—C27—C28	121.1 (6)	C17—C18A—H18A	109.6
C23—C28—C27	119.2 (6)	C17—C18A—H18B	109.6
C23—C28—C29	119.2 (5)	C19A—C18A—H18A	109.6
C27—C28—C29	121.6 (5)	C19A—C18A—H18B	109.6
C20—C29—C28	119.8 (5)	H18A—C18A—H18B	108.1
C22—O4—H4O	109.5	C17—C18B—H18C	106.8
C25—O5—H5O	109.5	C17—C18B—H18D	106.8
C2—C1—H1	119.3	C19B—C18B—H18C	106.8
C6—C1—H1	119.3	C19B—C18B—H18D	106.8
C1—C2—H2	120.4	H18C—C18B—H18D	106.6
C3—C2—H2	120.4	C18A—C19A—H19A	109.5
C2—C3—H3	119.1	C18A—C19A—H19B	109.5
C4—C3—H3	119.1	C18A—C19A—H19C	109.5
C3—C4—H4	120.6	H19A—C19A—H19B	109.5
C5—C4—H4	120.6	H19A—C19A—H19C	109.5
C4—C5—H5	119.5	H19B—C19A—H19C	109.5
C6—C5—H5	119.5	C18B—C19B—H19D	109.5
N1—C7—H7A	108.5	C18B—C19B—H19E	109.5
N1—C7—H7B	108.5	C18B—C19B—H19F	109.5
C6—C7—H7A	108.5	H19D—C19B—H19E	109.5
C6—C7—H7B	108.5	H19D—C19B—H19F	109.5
H7A—C7—H7B	107.5	H19E—C19B—H19F	109.5
N1—C8—H8A	108.3	C20—C21—H21	120.4
N1—C8—H8B	108.3	C22—C21—H21	120.3
C9—C8—H8A	108.3	C23—C24—H24	120.0
C9—C8—H8B	108.3	C25—C24—H24	120.0
H8A—C8—H8B	107.4	C25—C26—H26	120.8
C8—C9—H9A	109.0	C27—C26—H26	120.8
C8—C9—H9B	109.0	C26—C27—H27	119.5
C10—C9—H9A	109.0	C28—C27—H27	119.5
C10—C9—H9B	109.0	C20—C29—H29	120.1
H9A—C9—H9B	107.8	C28—C29—H29	120.1
			
O1—S1—C20—C21	−41.9 (6)	C12—C13—C14—C15	−173.9 (7)
O1—S1—C20—C29	137.6 (5)	N1—C16—C17—C18A	−144.5 (14)
O2—S1—C20—C21	77.2 (5)	N1—C16—C17—C18B	175.9 (11)
O2—S1—C20—C29	−103.3 (5)	C16—C17—C18A—C19A	−167.5 (18)
O3—S1—C20—C21	−163.2 (5)	C16—C17—C18B—C19B	156.7 (19)
O3—S1—C20—C29	16.3 (6)	S1—C20—C21—C22	177.5 (5)
C7—N1—C8—C9	−61.7 (8)	S1—C20—C29—C28	−177.4 (4)
C8—N1—C7—C6	−64.9 (7)	C21—C20—C29—C28	2.1 (9)
C7—N1—C12—C13	68.9 (7)	C29—C20—C21—C22	−2.0 (10)
C12—N1—C7—C6	54.5 (7)	C20—C21—C22—O4	179.0 (6)
C7—N1—C16—C17	178.5 (6)	C20—C21—C22—C23	−0.8 (10)
C16—N1—C7—C6	175.0 (6)	O4—C22—C23—C24	2.2 (9)
C8—N1—C12—C13	−169.1 (6)	O4—C22—C23—C28	−176.5 (5)
C12—N1—C8—C9	176.5 (6)	C21—C22—C23—C24	−177.9 (6)
C8—N1—C16—C17	57.9 (8)	C21—C22—C23—C28	3.3 (9)
C16—N1—C8—C9	55.1 (8)	C22—C23—C24—C25	−177.2 (6)
C12—N1—C16—C17	−61.0 (8)	C22—C23—C28—C27	177.5 (6)
C16—N1—C12—C13	−48.0 (8)	C22—C23—C28—C29	−3.1 (8)
C2—C1—C6—C5	0.2 (10)	C24—C23—C28—C27	−1.3 (9)
C2—C1—C6—C7	176.1 (9)	C24—C23—C28—C29	178.1 (5)
C6—C1—C2—C3	−0.0 (16)	C28—C23—C24—C25	1.5 (9)
C1—C2—C3—C4	0.3 (15)	C23—C24—C25—O5	−179.6 (5)
C2—C3—C4—C5	−0.9 (18)	C23—C24—C25—C26	0.4 (8)
C3—C4—C5—C6	1.1 (17)	O5—C25—C26—C27	177.5 (6)
C4—C5—C6—C1	−0.8 (14)	C24—C25—C26—C27	−2.5 (10)
C4—C5—C6—C7	−176.7 (9)	C25—C26—C27—C28	2.7 (10)
C1—C6—C7—N1	88.7 (9)	C26—C27—C28—C23	−0.8 (9)
C5—C6—C7—N1	−95.5 (9)	C26—C27—C28—C29	179.8 (4)
N1—C8—C9—C10	−163.2 (7)	C23—C28—C29—C20	0.4 (7)
C8—C9—C10—C11	176.1 (8)	C27—C28—C29—C20	179.8 (4)
N1—C12—C13—C14	−171.3 (6)		

Symmetry codes: (i) −*x*+1, −*y*, −*z*; (ii) *x*+1/2, *y*, −*z*+1/2; (iii) *x*−1/2, *y*, −*z*+1/2; (iv) −*x*+3/2, *y*−1/2, *z*; (v) −*x*+1, *y*−1/2, −*z*+1/2; (vi) −*x*+1/2, *y*−1/2, *z*; (vii) *x*+1/2, −*y*+1/2, −*z*.

### Hydrogen-bond geometry (Å, °)

**Table:**

*D*—H···*A*	*D*—H	H···*A*	*D*···*A*	*D*—H···*A*
O4—H4O···O2^i^	0.82	1.94	2.758 (6)	172
O5—H5O···O3^iii^	0.82	1.87	2.623 (6)	153

Symmetry codes: (i) −*x*+1, −*y*, −*z*; (iii) *x*−1/2, *y*, −*z*+1/2.

## References

[bb1] Burla, M. C., Camalli, M., Carrozzini, B., Cascarano, G. L., Giacovazzo, C., Polidori, G. & Spagna, R. (2003). *J. Appl. Cryst.* **36**, 1103.

[bb2] Burnett, M. N. & Johnson, C. K. (1996). *ORTEPIII* Report ORNL-6895. Oak Ridge National Laboratory. Tennessee, USA.

[bb3] Higashi, T. (1995). *ABSCOR* Rigaku Corporation, Tokyo, Japan.

[bb4] Mizuguchi, J., Sato, Y., Uta, K. & Sato, K. (2007). *Acta Cryst.* E**63**, o2509–o2510.

[bb5] Nash, R. J., Grande, M. L. & Muller, R. N. (2001). * Proceedings of the 7th International Conference on Advances in Non-Impact Printing Technology*, pp. 358–364.

[bb6] Rigaku (2006). *PROCESS-AUTO* and *CrystalStructure* Rigaku Corporation, Tokyo, Japan.

[bb7] Sato, Y., Uta, K. & Mizuguchi, J. (2009). *Acta Cryst* E**65**, o321. 10.1107/S1600536809001056 PMC2968364 21581926

[bb8] Sheldrick, G. M. (2008). *Acta Cryst.* A**64**, 112–122. 10.1107/S0108767307043930 18156677

[bb9] Uta, K. & Mizuguchi, J. (2009). *Acta Cryst* E**65**, o320. 10.1107/S1600536809000178 PMC2968190 21581925

[bb10] Uta, K., Sato, Y. & Mizuguchi, J. (2009). *Acta Cryst.* E**65**, o319. 10.1107/S1600536809001329 PMC2968198 21581924

